# A Case of Twin Pregnancy Simulating Fibroid Tumors

**Published:** 1892-04

**Authors:** Leslie Martin

**Affiliations:** Baldwinsville, New York; Bureau of Obstetrics, I. H. A.


### A CASE OF TWIN PREGNANCY SIMULATING FIBROID TUMORS.

LESLIE MARTIN, M. D., BALDWINSVILLE, NEW YORK.

(Bureau of Obstetrics, I. H. A.)

Mrs. O. C., æt. thirty-five; medium stature and build; black hair and eyes; weight one hundred and twelve pounds; menses always regular, and normal in color and quantity; married January 25th, 1888, and menses regular until June 4th; about the middle or last of June menses appeared again; and last of June in the morning she began to be dizzy, and everything smelled badly all day; she also began to be tired and feeble; there was but little nausea through the whole period of gestation. About the middle of July the menses appeared, about two hours, quite severe, and she called upon me at my office on the 18th; I gave her Puls. On the 21st she had nausea from the cooking and smelling of food; gave Colch.$^{21m}$, and from this date to August 2d gave indicated remedies as symptoms arose.

At this period she said there was a bunch near the navel on the right side, and it was then called a tumor or pregnancy. About the middle of August she detected a tumor on the left side, low in the groin, and she had a hæmorrhage at this time, filling vessel nearly half full, and had two or three other attacks of hæmorrhage afterward. Had hæmorrhage always after riding or a walk, and had no more hæmorrhages after she quit walking or riding.

About the 20th of September, 1888, she called upon Dr. Van de Warker, of Syracuse, an experienced gynæcologist of the old school. Upon examination he said that she had a tumor and it was a fibroid; also that she was pregnant in his opinion. His advice was, the only consideration is your safety. "I do not care," he said, with a snap of his finger, "*that* for the fœtus, and I am no Catholic and have no Catholic superstitions," and said that her only safety demanded that he take away the fœtus. She said to him, "Why not take away the tumor?" He said,

"I cannot take away the tumor without destroying part of the womb, and that would grow up so that the child could not be delivered," and he said the only alternative was then Cæsarean section.

At this stage he gave her a very formidable prognosis; she called again at his office in about a week, and he made another very thorough examination and was confirmed in his opinion, and he advised an immediate removal of the fœtus under consultation. The next morning she went to Clifton Springs Sanitarium and was examined by Mrs. Dr. Gault, and she agreed with Dr. Van de Warker that there was a fibroid tumor with pregnancy. Also that she had a polypus, and the case was so interesting that she wanted the opinion of the faculty in the case. The next morning four of them, besides Dr. Gault, made the examination and agreed in the diagnosis; and Mrs. Dr. Gault, then and there, removed a small jelly-like mass, placed it in the palm of her hand, passed it around for inspection, and claimed that it partly obstructed the passage.

The patient said that she had no more hæmorrhage after this time, and also said that she was very sore all over her abdomen on account of the examination. She returned again in six weeks for examination, and there was no change in their diagnosis and no signs of life, and they advised no meddling and wait results. Her very large condition caused her relatives and friends much anxiety, and the prognosis that had been given in her case by specialists.

She being at home on the 10th of September, I was called by her husband to give her case a very careful examination and study. I first examined her carefully for sounds of fœtal heart with stethoscope, and could plainly hear the heart's sound; also the placental murmur. I then told her that I was satisfied that she was pregnant; but with the advice that if she would keep out of the specialist's hands and wait until she felt life that would prove my diagnosis. About the last of October she said that she felt life. Prior to October the examination of the other physician corroborated my diagnosis. At this date she said that she wished to ask many questions about her condition, and her prospects of a favorable or unfavorable termination. I showed her the large plates of tumors in Martin & Magryer's *Atlas of Gynæcology*, and explained to her what effect the tumors would have upon the development of the womb, particularly their location, whether internal, external, or within the walls of the uterus She fully decided to wait and see what nature would do for her. At intervals from September 21st to 28th she received Puls.$^{2c}$. From October 22d to 25th, symptoms called for Sep.$^{2c}$. On October 25th for bloating, very full after eating, gave Lyc.$^{2c}$. October 30th she was attacked with intense itching all over her body, for which she received one dose Psorin$^{m}$. The itching recurred November 13th, for which she received one more dose Psorin$^{m}$, with no further recurrence of the itching,

December 6th, gave her Caulo., for aid in labor; February 26th, gave her three powders of Puls.$^{20}$, as she was nearing time for labor. I kept her steadily upon a vegetable diet. On March 6th, 1889, at eleven A. M. summoned to see Mrs. O. C. She said that she had some bearing-down pain this morning, and about two A. M. she felt something pass from her, and she flowed some bright red blood for a short time.

She says for the past week she has discharged a dark mucus; also, that the action of the child has not been so strong, and says for the past three or four days her abdomen has been so hard. She says that she feels well in every respect, and all of the secretions have been normal. She says that her right leg swelled some. She showed me the vessel, and I saw a clot about three inches long by about one inch in diameter, and perfectly cylindrical, color dark and firm, resisting breaking down. Also saw some bright blood on napkin.

I then told her she had better be examined, and I would try to learn the condition and relation of the organs and parts. In careful examination upon her back and left side, also standing, I found the pelvis clear, and could readily feel the distended womb against the symphysis pubis, also found the neck of the womb in its normal position, pointing toward the point of the coccyx; the neck was soft and flabby, and projected like a nipple; my finger readily passed into the os uteri up to the internal os, which was firmly contracted, and on removing finger it was covered with a thick bloody white mucus, no odor, and a very small dark clot adhered to my hand. On careful palpation and auscultation with the naked ear, I could clearly detect the sound of the foetal heart on the right side of the abdomen. On inspection I found the abdomen very large and prominent, and not developed equally. There was no central flat space as we usually see in twin pregnancy. There was a tumor as large as the fist projecting from the womb in the left lower region of abdomen, and another hard, prominent tumor near fundus of the womb, on the right of the mesial line of abdomen, also another as large as the fist in the right side of the abdomen, on a line with the navel. The rest of the abdominal tumor was smooth and not modulated, but very hard and unyielding, and on palpation I could detect a weak movement of the child in the fundus of the womb on the left of the mesial line. The abdominal tumor is and has been located centrally all the period of gestation.

As I had never seen such a case, I gave her my reason why I did not examine her earlier was that, as she had been examined by such competent specialists, that I should rest quietly and await developments.

March 7th. Messenger came at two A. M., saying that Mrs. O. C. wished my services, as labor was at hand. On examination found the os dilating and pain regular, and everything progressing satisfactorily.

At this stage of the labor I said to the nurse that I would lie down, and when the pain became very active to call me. The nurse called me at four A. M., and found pain regular and labor progressing finely, and at seven A. M. she was delivered of a living male child, and before I could ligate the cord, she says, "Oh! dear, what a sharp pain I have," and I promptly examined her and found another child in utero; this child, a male, was born in about twenty minutes, dead, and showed all the external appearances of having been dead for about a week, the tissues around the eyes, mouth, and nose, gave full evidence of this; she had severe post-partum hæmorrhage, which was controlled with China.[2c].

On extracting the placenta and examining her carefully with one hand in the cavity of the womb and the other external, no tumors could be found, and on examination, after two months had elapsed, no tumors could be found.

She made a very slow recovery, but in a few weeks she began to recover rapidly, and she afterward nursed her child and became strong and well. I learned from the nurse afterward that the cause of death of the twin was that she had a very severe quarrel with her Irish nurse about one week before March 6th, and that on March 6th she showed me the clot and complained that her abdomen was so hard. The dead child was a perfect counterpart in features of the living one, and just as fully developed, and weighed the same as the living child.

In searching obstetric works carefully for such cases as narrated, we find but very few of those conditions mentioned. Dupaul, and one other author, whose name I have forgotten, alludes to such cases, and are classed as cases of unequal development of the walls of the uterus; this unequal development of the muscular walls in this case would aid to explain the severe hæmorrhage. In this condition the muscular walls of the uterus could not contract equally.

